# Liposomal Targeting of Prednisolone Phosphate to Synovial Lining Macrophages during Experimental Arthritis Inhibits M1 Activation but Does Not Favor M2 Differentiation

**DOI:** 10.1371/journal.pone.0054016

**Published:** 2013-02-28

**Authors:** Wouter Hofkens, Rik Schelbergen, Gert Storm, Wim B. van den Berg, Peter L. van Lent

**Affiliations:** 1 Rheumatology Research and Advanced Therapeutics, Department of Rheumatology, Radboud University Medical Centre, Nijmegen, The Netherlands; 2 Department of Pharmaceutics, Utrecht Institute for Pharmaceutical Sciences, Utrecht University, Utrecht, The Netherlands; University of Leuven, Rega Institute, Belgium

## Abstract

**Background:**

To determine the effects of liposomal targeting of prednisolone phosphate (Lip-PLP) to synovial lining macrophages on M1 and M2 polarization in vitro and during experimental arthritis.

**Material and Methods:**

Experimental arthritis (antigen and immune complex induced) was elicited in mice and prednisolone containing liposomes were given systemically. Synovium was investigated using microarray analysis, RT-PCR and histology. Bone–marrow macrophages were stimulated towards M1 using LPS and IFNγ before treatment by PLP-liposomes. M1 and M2 markers were determined using RT-PCR.

**Results:**

Microarray analysis of biopsies of inflamed synovium during antigen induced arthritis (AIA) showed an increased M1 signature characterized by upregulation of IL-1β, IL-6 and FcγRI starting from day 1 and lasting up until day 7 after arthritis induction. The M2 signature remained low throughout the 7 day course of arthritis. Treatment of AIA with intravenously delivered Lip-PLP strongly suppressed joint swelling and synovial infiltration whereas colloidal gold containing liposomes exclusively targeted the macrophages within the inflamed synovial intima layer. *In vitro* studies showed that Lip-PLP phagocytosed by M1 macrophages resulted in a suppression of the M1 phenotype and induction of M2 markers (IL-10, TGF-β, IL-1RII, CD163, CD206 and Ym1). *In vivo*, Lip-PLP treatment strongly suppressed M1 markers (TNF-α, IL-1β, IL-6, IL-12p40, iNOS, FcγRI, Ciita and CD86) after local M1 activation of lining macrophages with LPS and IFN-γ and during experimental AIA and immune complex arthritis (ICA). In contrast, M2 markers were not significantly upregulated in antigen-induced arthritis and down regulated in immune complex arthritis.

**Conclusion:**

This study clearly shows that systemic treatment with PLP-liposomes selectively targets synovial lining macrophages and inhibits M1 activation. In contrast to *in vitro* findings, PLP-liposomes do not cause a shift of synovial lining macrophages towards M2.

## Introduction

Synovial lining macrophages play a crucial role in the onset and maintenance of joint inflammation during arthritis [1], [2]. Previous studies have shown that their selective elimination with clodronate-liposomes prior to induction or during established experimental arthritis resulted in largely diminished synovial inflammation [3], [4]. Although the activation stage of macrophages is very versatile, various subpopulations have been defined reflecting stadia of polarization. Classically activated macrophages are induced by combined stimulation with lipopolysaccharide (LPS) and interferon gamma (IFN-γ) and these macrophages express a unique set of genes giving rise to a pro-inflammatory phenotype. Characteristically, these cells produce cytokines like TNF-α, IL-1β, IL-6 and IL-12 in high amounts and upregulate MHC-II and CD86, which facilitate antigen presentation [5], [6]. The pro-inflammatory activation state of macrophages can be further enhanced through the high affinity receptor FcγRI in response to immune-complexes [7]. Furthermore, classically activated macrophages produce reactive oxygen species like nitric oxide (NO) via nitric oxide synthase 2 (NOS2/iNOS) and stimulate T-cells towards a Th1 or Th2 phenotype [8].

More recently, it has been described that macrophages can also be alternatively activated *in vitro*, typically by IL-4, to induce a macrophage with an anti-inflammatory phenotype [7]. These cells express cytokines such as IL-10, with known anti-inflammatory properties and upregulate arginase 1 which inhibits NO production. They also suppress antigen presentation molecules and T-cell proliferation.

Classically activated, pro-inflammatory macrophages and alternatively activated, anti-inflammatory macrophages are now generally referred to as M1 and M2 macrophages respectively. More recently, several studies have indentified these subsets of macrophages in animal models. Typically, M1 macrophages are associated with infection [9], inflammation [10] and tissue injury [11]. M2 macrophages are suppressed within these models, but may have a role in the resolution of inflammation and in wound repair [11].

Although glucocorticoids are known since long for their strong inhibition of inflammation, their effect on subsets of macrophages is only recently emerging. *In vitro* studies performed with human and murine monocytes showed that glucocorticoids can drive monocytes towards an M2-like phenotype characterized by expression of CD163, a strong marker for M2 macrophages [8], [12]. In line with that, monocytes from healthy volunteers showed upregulation of CD163 after relatively high doses of intravenous glucocorticoids [13].

Glucocorticoids can be targeted to inflamed knee joints more effectively by systemic intravenous injection within long circulating ‘stealth’ liposomes during experimental arthritis [8], [14]. Recently, we found that intravenous liposomal delivery of glucocorticoids greatly improved its potency and a single injection strongly inhibited knee joint inflammation in experimental arthritis [15]–[17]. The strong effect on inhibition of joint inflammation may be due to alteration of the macrophage phenotype within the lining layer.

The aim of this study was to determine the effect of the liposomally delivered glucocorticoid prednisolone phosphate (Lip-PLP) on M1/M2 polarization of macrophages within the synovial intima layer. For this, we studied gene expression of various M1 and M2 markers in the inflamed synovium during immune-complex induced arthritis (ICA) and antigen-induced arthritis (AIA). In ICA, the synovium is activated by immune complexes whereas in AIA, activation is driven by both immune complexes and T cells. As in the arthritis models the synovium is highly infiltrated with leukocytes, we also studied the effect of Lip-PLP in a model in which the synovium was activated towards an M1 phenotype with LPS and IFN-γ by local injection into the knee joint, which did not result in synovial infiltration. Additionally, we studied the direct effect of Lip-PLP on M1 activated bone marrow derived macrophages *in vitro*. Our results show that PLP-liposomes target synovial intima cells and inhibit M1 macrophages but, in contrast to *in vitro* studies, do not skew them to a more M2 phenotype.

## Materials and Methods

### Ethics statement

All *in vivo* studies were carried out in strict accordance with the recommendations in the Guide for the Care and Use of Laboratory Animals of the Dutch national legislation. The protocol was approved by the local Committee on the Ethics of Animal Experiments of the Radboud University Nijmegen (Permit Number: RU-DEC 2006-182). All surgery was performed under 2,5% isoflurane with N_2_O/O_2_ anesthesia, and all efforts were made to minimize suffering.

### Liposome preparation

Liposomes were prepared as described previously [17], using a lipid formulation of dipalmitoyl phosphatidylcholine (DPPC, Lipoid GmbH, Ludwigshave, Germany), PEG 2000-distearoyl phosphatidylethanolamine (DSPE) and cholesterol (Sigma Chemical Co., Poole, UK) in a molar ratio of 1.85∶0.15∶1.0. These lipids were dissolved in ethanol which was then evaporated from a round-bottom flask to create a lipid film. The lipid film was hydrated in a solution of 100 mg/ml prednisolone disodium phosphate (PLP, Bufa, Uitgeest, the Netherlands) in water to create liposomal PLP. Single unilamellar vesicles were obtained by filtering the liposomal dispersion multiple times through polycarbonate filter membranes decreasing in pore diameter until the liposomes had a mean diameter in the range of 90–110 nm with a polydispersity of <0.2. Mean particle size was determined by dynamic light scattering with a Malvern 4700 system (Malvern ltd., Malvern, UK). Unencapsulated PLP was removed by dialysis against 0.9% phosphate buffered saline using Slide-A-Lyzer dialysis cassettes with a molecular weight cut-off of 10,000 (Pierce, Rockford, IL, USA). Encapsulation dose of PLP was determined by extracting the aqueous phase from liposomal preparations with chloroform. The aqueous phase after extraction was used for determining the PLP content using high performance liquid chromatography using a mobile phase acetonitril-water with pH of 2, connected to a UV-detector, which was set at 254 nm. Both prednisolone and its phosphate ester could be measured in one single run. Liposomal preparations contained around 5 mg/ml PLP (slightly varying between batches) and an average of 60 µmol phospholipid. Liposomes containing colloidal gold were prepared in a similar manner except for the hydration step of the lipid film, which was performed with a freshly prepared tetrachloroaurate solution in citrate buffer. Colloidal gold was formed after sizing the liposomes at 4°C and subsequently incubating the liposomes at 37°C. The non-encapsulated gold was removed by eluting the preparation on a Sephacryl S1000-SF column (Pharmacia, Uppsala, Sweden).

### Animals

Mice (C57Bl/6, female) were purchased from Elevage-Janvier (Le Genest Saint Isle, France) and were housed in the central animal Lab in Nijmegen, The Netherlands in filter-top cages and fed a standard diet and water ad libitum.

### Antigen-induced arthritis (AIA)

AIA was induced as described previously [18]. Briefly, mice at an age of 8–12 weeks were immunized with 100 µg methylated bovine serum albumin (mBSA, Sigma-Aldrich, St Louis, USA), emulsified in Freund's complete adjuvant (Difco Laboratories, Detroit, USA) which was injected into the flanks and the footpad of the forelegs of the mice. Heat-killed *Bordetella pertussis* (RIVM, Bilthoven, the Netherlands) was administered intraperitoneally as an additional adjuvant. Two subcutaneous booster injections with in total 50 µg mBSA/Freund's complete adjuvant were given in the neck region 1 week after the initial immunization. At week 3 after the initial immunization, AIA was induced by intra-articular injection of 60 µg of mBSA in 6 µl saline into the knee joints. Mice were treated at day 3 after induction of the AIA by intravenous injection of liposomal PLP or free PLP (both 10 mg/kg), or saline as a control. Mice were sacrificed at day 1 or day 5 after treatment and tissues were isolated hereafter.

### Immune-complex arthritis (ICA)

Immune-complex arthritis (ICA) was passively induced in knee joints of mice as described previously [19] by direct intra-articular injection of 3 µg of lysozyme in 6 µl saline into the knee joints of mice that were intravenously injected anti-lysosyme antibodies 24 hours earlier. Mice were treated with intravenously injected liposomal PLP (10 mg/kg) or saline at day 1 after induction of the ICA. Twenty-four hour after treatment, mice were sacrificed and tissues were isolated.

### Local activation of the synovial lining in the knee joint

Naïve mice were injected intra-articularly with 6 µl saline containing interferon gamma (IFN-γ, 100 ng) and *Escherichia coli* lipopolysaccharide (LPS, 1 µg) to induce the M1 phenotype in macrophages within the synovial lining. These mice and naïve control mice were treated 24 hours thereafter by intra-articular injection of 6 µl of Lip-PLP (50 µg) or saline as a control. Twenty-four hours after treatment, mice were sacrificed and tissues were isolated.

### Measurement of ^99M^Technetium-uptake

Uptake of ^99M^Technetium (Tc) was measured as described previously [20] to determine the swelling of the knee joint that occurs as a consequence of inflammation. Mice were sedated with 4.5% chloral hydrate and intraperitoneally injected with 20 µCi of ^99M^Tc. After 30 minutes, the amount of radioactivity was determined by external gamma counting. Knee joint swelling was expressed as the ratio of ^99M^Tc uptake in the right (R) and left (L) knee joint of mice with an unilaterally induced arthritis in the right knee joint. Right-left ratios >1.1 were taken to indicate significant swelling of the right knee joint.

### Sacrifice and tissue collection

Mice were sacrificed by cervical dislocation and arthritic knee joints were isolated and fixed in 10% formalin for 4 days for histological analysis. For RNA isolation, biopsies with a diameter of 3 mm were punched out of the synovium from both the lateral and medial side of the arthritic knee joints as described previously [21] and were stored in liquid nitrogen until RNA isolation.

### Histology

After fixation, total knee joints were decalcified in 5% formic acid and thereafter embedded in paraffin. Standard frontal sections of 7 µm were mounted on superfrost slides (Menzel-Gläser, Braunschweig, Germany) and stained with hematoxylin and eosin (HE). The severity of joint inflammation was determined as described previously [22], by scoring the amount of cellular infiltration into the synovium using an arbitrary scale (0–3), for three representative knee joint sections for each mouse (5 mice for each treatment group). Scoring was performed in a blinded manner by two independent observers: 0, no cells; 1, mild cellularity; 2, moderate cellularity; 3, maximal cellularity.

### Visualization of gold-liposomes

To visualize the uptake of gold-containing liposomes, knee joints were removed from mice 24 hours after treatment with gold-liposomes and were decalcified in ECTA/PVP (polyvinylpyrrilodine) in TRIS buffer for 2 weeks. Knee joints were then frozen in liquid nitrogen and sections were cut on a cryostat (Microm HM500M, Waldorf, Germany) and mounted on Superfrost microscopic slides (Menzel Gläser, Germany). Silver enhancement of colloidal gold was performed with Sigma silver enhancement kit (Sigma, St. Louis, MO, USA) and terminated by incubating with a 0.5% sodium thiosulphate solution in distilled water. Sections were counterstained with hematoxylin.

### Macrophage culture

To obtain murine macrophages *in vitro*, bone marrow cells from femoral shafts of C57Bl/6 mice were cultured in DMEM medium (Invitrogen, Basel, Switzerland), supplemented with 10% FCS, antibiotics and 10 ng/ml M-CSF (R&D systems). Medium was refreshed every 3 days. Bone marrow macrophages were incubated with 10 ng/ml IFN-γ and 100 ng/ml *Escherichia coli* LPS (Sigma-Aldrich) for 24 hours to induce M1 macrophages and subsequently treated with 10 µg/ml Lip-PLP for another 24 hours. At day 7 of the macrophage culture (24 hours after M1 induction and treatment), cells were submersed in Trizol reagent (Invitrogen) for RNA isolation or scraped loose for flow cytometry. Culture supernatant was stored at −20°C until measurement of cytokine levels.

### Measurement of cytokine levels

Cytokine levels of TNF-α, IL-1β, IL-6 and IL-12 were measured in macrophage culture supernatant using Luminex multianalyte technology, (Bio-Rad Laboratories, Hercules, USA) according to the manufacturer's instructions. Protein levels were calculated from a standard curve of known cytokine concentrations. Data analysis was performed using Bio-Plex Manager software (Bio-Rad Laboratories).

### Flow cytometry

Surface levels of CD86 were measured by flow cytometry. Cells were counted, washed and incubated with PE-labeled rat anti-mouse CD86 antibody (BD Pharmingen) for one hour. After washing, labeling of the cells was measured by flow cytometry using a FACSCalibur (BD Biosciences). Mean fluorescence intensity (MFI) was corrected against a relevant isotype control staining.

### RNA isolation

RNA was isolated with a RNeasy kit (Qiagen, Venlo, the Netherlands). Isolated nucleic acids were treated with DNAse before being reverse transcribed into complementary DNA using oligo (dT) primers and MMLV reverse transcriptase.

### Microarray analysis

The microarray was performed as described previously [10], using Affymetrix oligonucleotide arrays. Generation of biotinylated complementary RNA and subsequent hybridization, washing and staining of oligonucleotide arrays (Affymetrix, Santa Clara, CA) were performed according to the Affymetrix Expression Analysis Technical Manual for 1-cycle amplification. The arrays were then scanned using a laser scanner (GeneChip Scanner; Affymetrix) and analyzed using Affymetrix GeneChip Operating Software (GCOS; version 1.4) according to the manufacturer's instructions. Gene expression relative to the house-keeping gene GAPDH for each time point during AIA is presented as fold change from expression levels of naïve mice (means of 3 mice per group).

### Quantitative reverse transcriptase-polymerase chain reaction (RT-PCR)

The RT-PCR was performed using the ABI Prism 7000 Sequence Detection system (Applied Biosystems) for quantification with SYBR Green and melting curve analysis. Primers were designed with Primer Express Version 2.0 (Applied Biosystems). PCR conditions were as follows: 2 minutes at 50°C and 10 minutes at 95 50°C, followed by 40 cycles of 15 seconds at 95°C and 1 minute at 60°C. Primer concentrations were 300 nM. All PCR's were performed in a total volume of 20 µl. Data are presented as expression levels relative to the house-keeping gene GAPDH (means of 5 mice per group).

### Statistical analysis

Differences between treatment groups was tested for statistical significance with Student's t-test using GraphPad Prism 5 software. Results are expressed as mean +/− S.D.

## Results

### Inflamed synovium strongly expresses a dominant M1 signature during the course of antigen-induced arthritis

**Figure 1:**
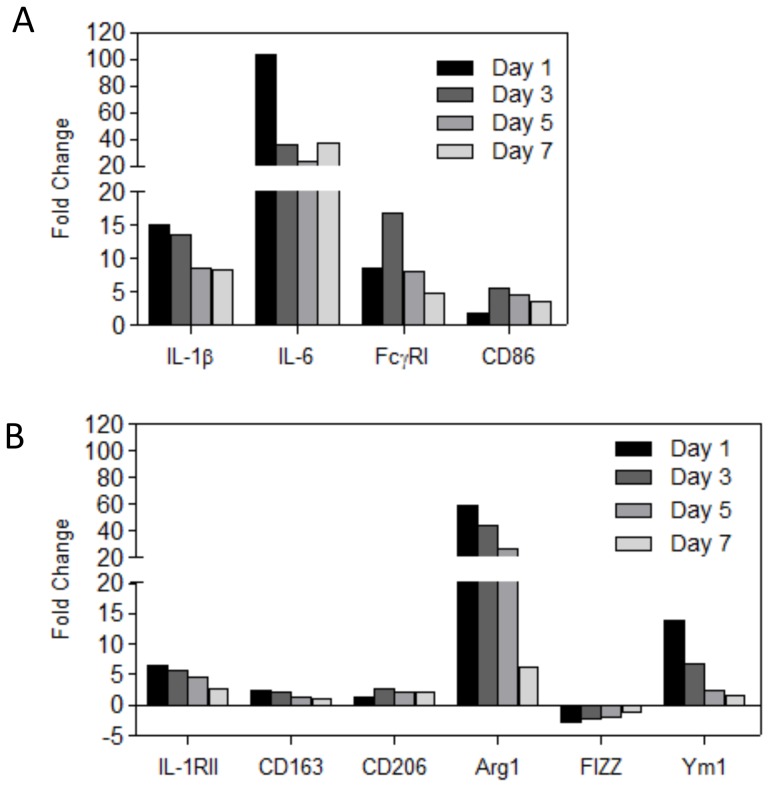
Expression of M1 and M2 markers within the inflamed synovium during AIA as determined by micro-array analysis. Gene expression was determined at day 1, 3,5 and 7 after induction of AIA. Fold increase of gene expression was compared to synovium of naïve mice. A: Expression of M1 markers (IL-1β, IL-6, FcγRI and CD86). B: Expression of M2 markers (IL-1RII, CD163, CD206, Arg1, FIZZ1 and Ym1). Note that expression of M1 markers is highly upregulated compared to M2 markers, with the exception of Arg1 and Ym1. Values are presented as the fold change in mean gene expression levels (relative to GAPDH) from mean gene expression levels of inflamed synovium (n = 8) compared to synovium drived from naïve mice (n = 3).

To characterize the expression of M1 and M2 markers in the inflamed synovium during experimental arthritis, we isolated messenger RNA from synovial biopsies in a standard manner [21] at various time points (days 1, 3, 5 and 7) after induction of antigen-induced arthritis (AIA). Gene expression in inflamed synovium was determined by microarray as described earlier [10] and compared with control synovium obtained from normal mouse knee joints. Microarray analysis showed that various M1 markers (IL-1β, IL-6, FcγRI and CD86) were strongly upregulated at day 1 after induction of arthritis up to day 7 (Fig. 1A). The majority of M2 markers (IL-1RII, CD163, CD206, Arg1 and Ym1) were also somewhat upregulated during the course of arthritis, although in lesser extent than the M1 markers, with the exception of Arg1 and Ym1 which were especially high at day 1 after induction of AIA (Fig. 1B). Altogether though, this suggests a shift towards a M1 signature in the inflamed synovium during AIA.

### Targeting the inflamed synovium with glucocorticoid liposomes strongly suppresses experimental arthritis

**Figure 2:**
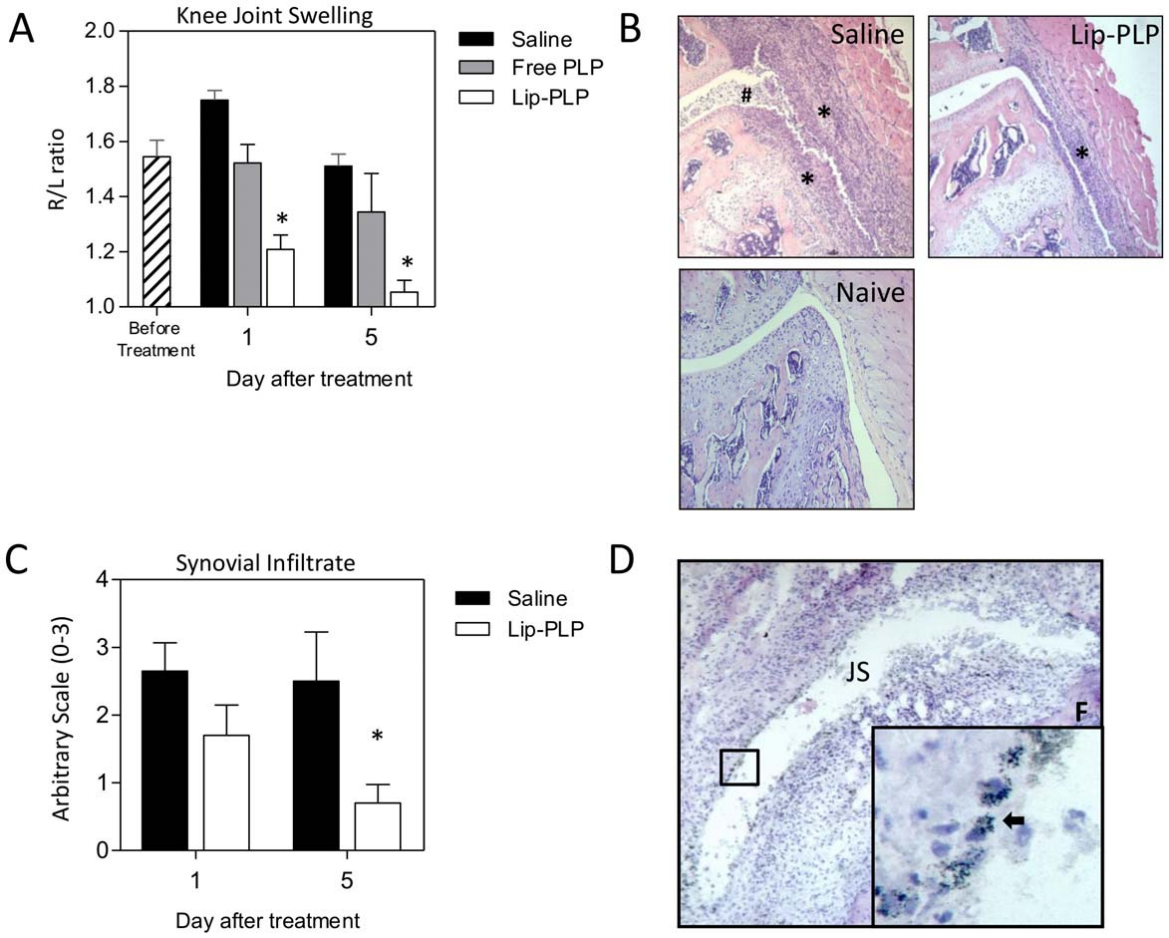
Liposomal targeting of PLP to the inflamed synovial lining strongly suppresses joint inflammation during AIA. A: Knee joint swelling as measured by ^99M^Tc-uptake is strongly suppressed after a single injection of Lip-PLP. B: Photomicrographs of frontal knee joint sections of mice with AIA at day 5 after treatment and naïve mice. Note that the inflammatory infiltrate is reduced in mice treated with Lip-PLP. Original magnification ×100, Asterisks points to synovial infiltrate, hash sign points to inflammatory exudates. C: Histological scoring of synovial infiltration at day 1 and day 5 after systemic treatment with Lip-PLP or saline. D: Silver staining of frontal knee joint sections of mice with AIA, treated by intravenous injection with gold-containing liposomes. Note that the silver staining of the gold particles is mostly observed within the synovial lining cells (arrows). Mice were treated at day 3 after induction of AIA. Values are the mean of 8 mice per group. Original magnification ×100; insert ×400. F = femur, JS = joint space. Statistical significance was determined by Student's t-test. * = P<0.05 compared to saline treatment.

Mice with established antigen-induced arthritis (AIA) expressing a strong M1 signature at day 3, were treated with a single intravenous injection of Lip-PLP (10 mg/kg) and showed a strong suppression of joint swelling as measured by ^99M^Tc-uptake by 74% within 1 day when compared to saline controls and by 61% when compared to free PLP treatment (Fig. 2A). At day 5 after treatment, Lip-PLP had almost completely suppressed joint swelling and histological examination of frontal knee joint sections showed a mean suppression of the synovial infiltrate of 29% at day 1 and of 80% at day 5 after treatment (Fig. 2B+C).

### Intravenous injection of gold-liposomes targets synovial lining macrophages

To determine whether the Lip-PLP formulation is directly targeted to macrophages in the synovial intima layer, we injected liposomes containing colloidal gold intravenously into mice with day 3 AIA. Silver enhancement staining of frontal sections of the inflamed knee joint showed that at day 1 after injection, the gold-laden liposomes were taken up by macrophages lying within the synovial intima (Fig. 2D) suggesting that these liposomes leave the bloodstream through the vessels lying just beneath the lining layer and then become directly engulfed by the intima macrophages. Type B synovial fibroblasts do not take up liposomes and may thus be less affected [23].

### Lip-PLP skews M1 macrophages towards an M2 phenotype *in vitro*

**Figure 3:**
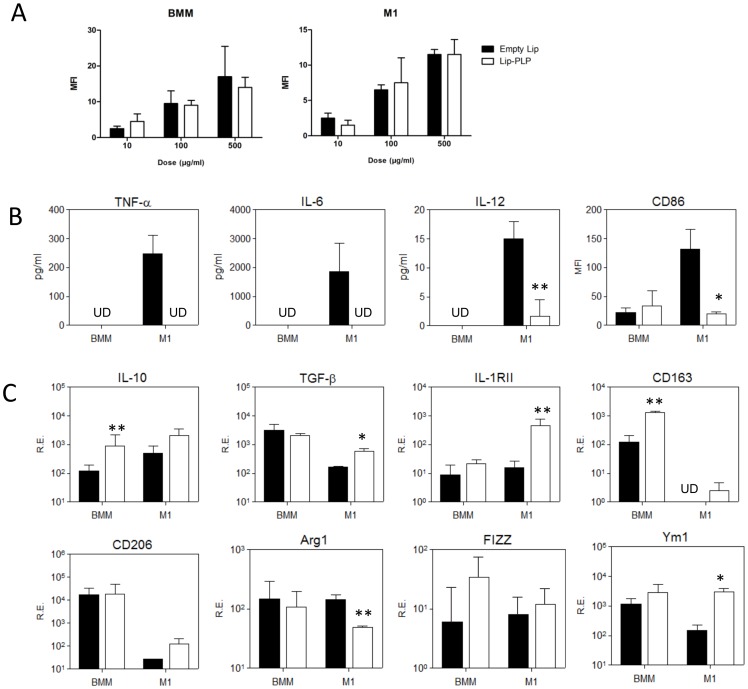
Effect of Lip-PLP on M1 macrophages *in vitro*. Cells and supernatant of bone-marrow macrophages (BMM) and M1 macrophages (stimulated with LPS and IFN-γ for 24 hours) were obtained 24 hours after treatment with Lip-PLP or saline. A: Uptake of fluorescent liposomes by BMM and M1 macrophages as measured by flow cytometry. Note that uptake of liposomes is dependent on the amount of liposomes but not on PLP content. B: Protein levels of M1 cytokines TNF-α, IL-6 and IL-12 within the supernatant and surface expression of M1 marker CD86 as determined by flow cytometry. C: Gene expression of M2 markers. RE = Relative Expression compared to values of GAPDH. Data are expressed as mean +/− S.D. UD = undetectable. Three independent experiments were performed. Statistical significance was determined by Student's t-test. * = P<0.05, ** = P<0.01 compared to saline treatment.

To study the direct effect of Lip-PLP on activated macrophages, we first investigated whether liposomal PLP may alter M1 macrophages into an M2 phenotype *in vitro*. Bone marrow-derived macrophages (BMMs) were stimulated towards an M1 type using IFN-γ (10 ng/ml) and LPS (100 ng/ml) for 24 hours and subsequently treated with Lip-PLP for another 24 hours. Liposomes were directly engulfed by non-stimulated macrophages and M1 macrophages as measured by flow cytometry of fluorescently labeled empty and PLP-liposomes (10, 100 and 500 µg/ml, Fig. 3A). PLP-liposomes did not cause cell death as measured by trypan blue uptake and by counting living cells and flow cytometry of apoptotic cells with 7-AAD staining (data not shown).

Cytokine and membrane markers reflecting the polarization status of M1 and M2 were measured by Luminex, flow cytometry and QPCR. Treatment of M1 macrophages with Lip-PLP strongly suppressed protein levels of M1 cytokines TNF-α (100%), IL-6 (100%), and IL-12 (91%) (Fig. 3B). Furthermore, Lip-PLP significantly suppressed the M1 status as represented by surface expression of CD86 by 82% (Fig. 3B).

To evaluate whether Lip-PLP treatment skews BMMs and M1 macrophages towards an M2 phenotype, we measured gene expression of various generally accepted M2 markers. In BMMs Lip-PLP treatment strongly upregulated mRNA levels of M2 associated genes IL-10 (7-fold) and CD163 (10-fold) (Fig. 3C). In M1 macrophages Lip-PLP treatment strongly upregulated mRNA levels of M2 associated genes IL-10 (3-fold), TGF-β (3-fold), IL-1RII (14-fold), CD163 (undetected in M1 macrophages), CD206 (5-fold) and Ym1 (12-fold) (Fig. 3C), indicating that Lip-PLP is capable of skewing both BMMs as well as M1 macrophages towards an M2 phenotype.

### Glucocorticoid liposomes suppress M1 activation but do not induce polarization of synovial lining macrophages towards M2

**Figure 4:**
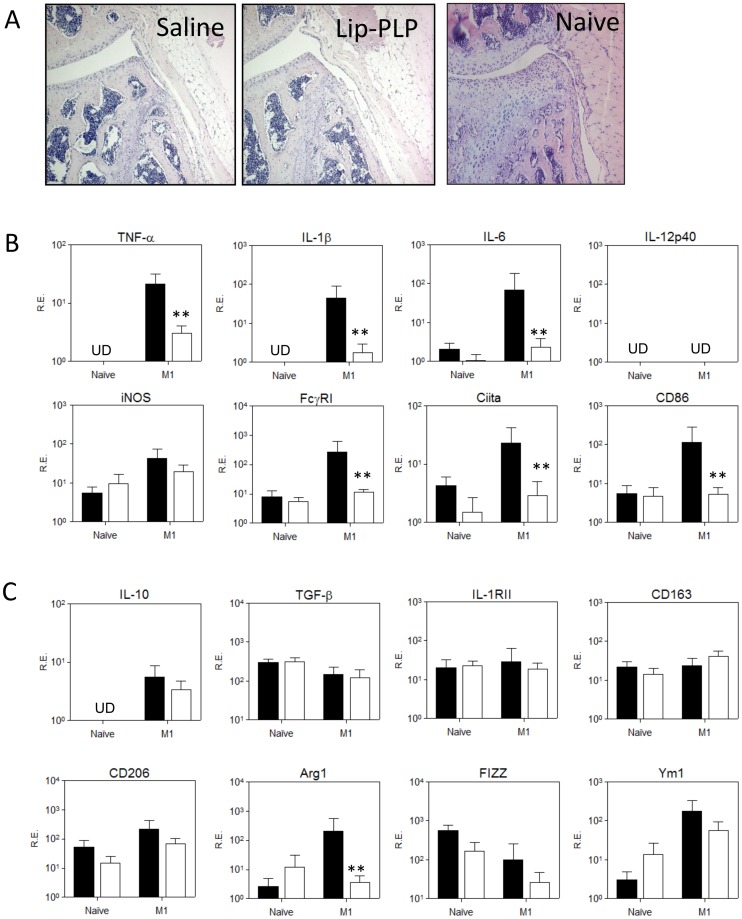
Effect of Lip-PLP on M1 and M2 marker expression within the synovium after local M1 activation. Macrophages of the synovial lining in the knee joints were activated towards M1 by intra-articular injection of LPS and IFN-γ for 24 hours and were subsequently treated by intra-articular injection of Lip-PLP or saline for 24 hours. A: Frontal knee joint sections of mice after local M1 activation and subsequent treatment with Lip-PLP or saline and naïve mice. B+C: Expression of M1 (B) and M2 (C) markers in the synovium. RE = Relative Expression compared to values of GAPDH. Values represent the mean +/− SD of eight mice. UD = undetectable. Statistical significance was determined by Student's t-test. * = P<0.05, ** = P<0.01 compared to saline treatment.

Next, we investigated whether intra-articular injection of Lip-PLP was able to alter M1 into an M2 signature in the synovial lining of the murine knee joint. We first induced an M1 signature in the lining macrophages by injection of LPS (1 µg) and IFN-γ (100 ng). At 24 hours thereafter, no significant cellular infiltrate of the synovium was found (Fig. 4A). However, synovial biopsies which included the intima layer showed high expression of mRNA levels of M1 type cytokines TNF-α, IL-1β and IL-6 (13-, 16- and 16-fold, respectively) and of M1 markers iNOS, Ciita and CD86 (10-, 8- and 12-fold, respectively) when compared to naïve synovium (Fig. 4B). Injection of Lip-PLP (50 µg) into the M1 knee joint strongly suppressed all the upregulated M1 type genes to levels not significantly different from those in naïve mice when measured at 24 hours thereafter (with the exception of iNOS). In contrast, expression of M2 markers IL-10, IL-1RII, CD163, CD206 and FIZZ1 was hardly changed by Lip-PLP treatment (with the exception of Arg1) (Fig. 4B). These results suggest that local injection of Lip-PLP inhibits M1 macrophages but does not induce polarization towards M2 macrophages.

### Systemic delivery of Lip-PLP during antigen induced arthritis suppresses the M1 synovial macrophage without altering the M2 phenotype within the inflamed synovium

**Figure 5:**
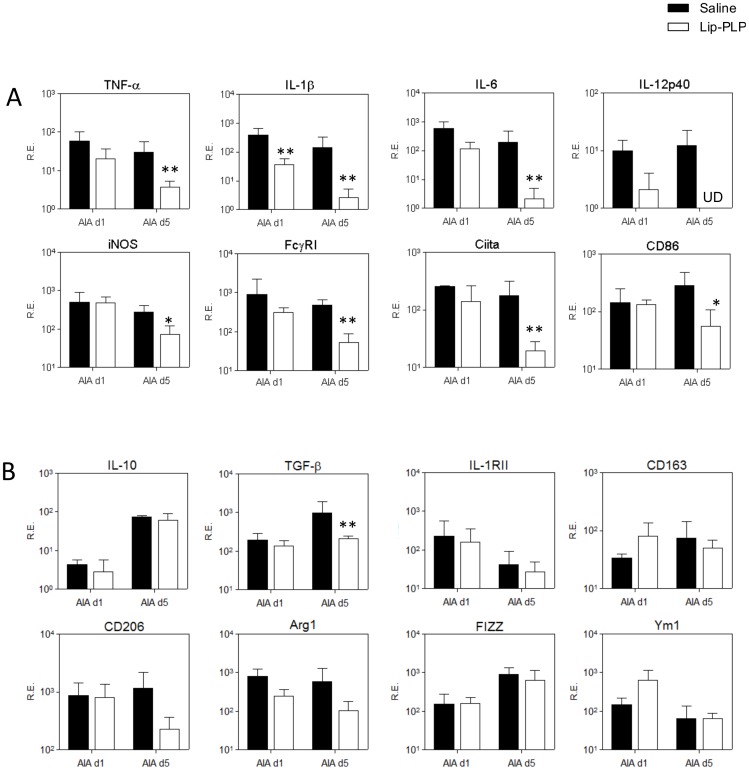
Effect of Lip-PLP on M1 and M2 marker expression within the synovium during AIA. A: Expression of M1 markers. B: Expression of M2 markers. Mice were treated by intravenous injection of Lip-PLP or saline at day 3 after induction of the AIA and synovial biopsies were obtained at day 1 and day 5 after treatment. RE = Relative Expression compared to values of GAPDH. Data are expressed as mean +/− SD of eight animals. UD = undetectable. Statistical significance was determined by Student's t-test. * = P<0.05, ** = P<0.01 compared to saline treatment.

To determine whether the M1 phenotype is suppressed in favor of M2 by systemic treatment with PLP-liposomes during experimental arthritis, we measured gene expression of various M1 and M2 markers in the synovium at day 1 and day 5 after systemic treatment with Lip-PLP of established AIA (day 3). Treatment with PLP-liposomes resulted in a rapid and strong down regulation of mRNA levels of M1 type cytokines TNF-α (8-fold), IL-1β (55-fold), IL-6 (94-fold) and IL-12 (levels not detected) at day 5 after treatment in the synovium (Fig. 5A). Additional genes reflecting M1 activation like FcγRI, Ciita and CD86, were also significantly suppressed at day 5 after treatment (9-, 10- and 6-fold, respectively), indicating a silencing of the M1 pattern by Lip-PLP (Fig. 5B). In contrast to M1, expression of M2 markers IL-10, IL-1RII, CD206, Arg1, CD163, FIZZ1 and Ym1 was not significantly downregulated, even at day 5 after treatment (with the exception of TGF-β), which does not point to a shift in phenotype from M1 to M2 after treatment with Lip-PLP (Fig. 5B).

### Systemic delivery of PLP-liposomes suppresses M1 activation during locally induced immune-complex arthritis (ICA)

As antigen-induced arthritis is largely driven by T- and B-cells, intravenous delivery of PLP-liposomes may alter systemic immunity, which contributes to the rapid and strong effects on joint inflammation. In order to investigate the direct effect of Lip-PLP accumulation in the lining on the joint inflammation in more detail, we finally tested a locally induced immune-complex arthritis which is not dependent on T- or B-cell immunity. This arthritis model is largely driven by macrophages in the knee joint in response to local application of antibody-complexes in the joint [19].

**Figure 6:**
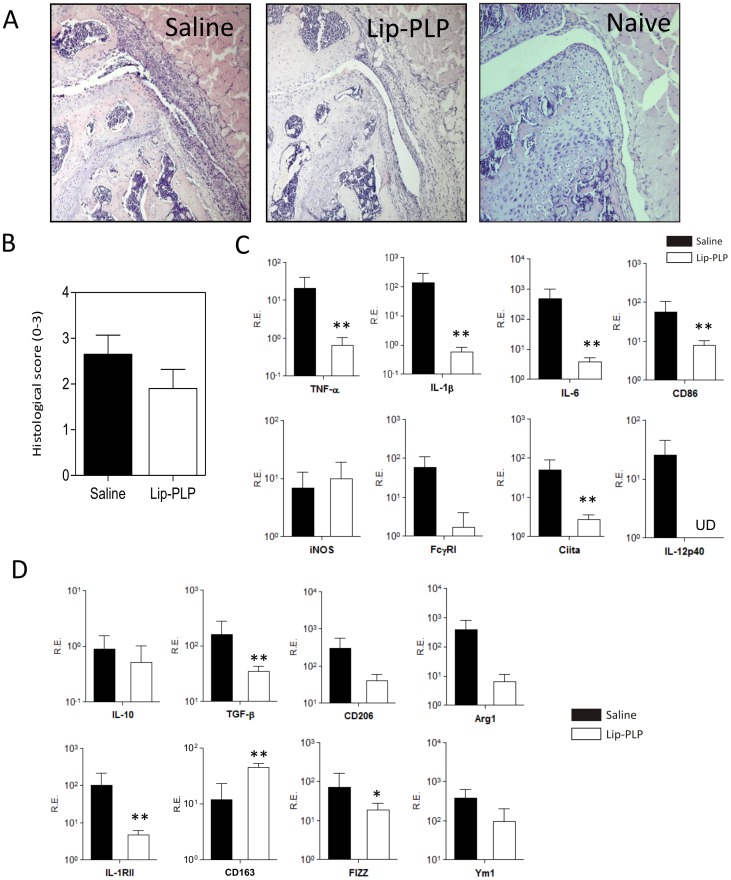
Effect of Lip-PLP on M1 and M2 marker expression within the synovium during ICA. A: Photomicrographs of frontal knee joint sections of mice with ICA at day 1 after treatment. B: Histological scoring of synovial infiltration mice with ICA at day 1 after treatment with saline or Lip-PLP. C+D: Expression of M1 (C) and M2 (D) markers in the synovium during ICA. RE = Relative Expression compared to values of GAPDH. Mice were treated at day 1 after induction of ICA and biopsies were obtained at day 1 after treatment. Data are expressed as mean +/− SD of eight animals. UD = undetectable. Statistical significance was determined by Student's t-test. * = P<0.05, ** = P<0.01 compared to saline treatment.

Similar to the AIA, systemic delivery of Lip-PLP inhibited synovial infiltration at day 1 after injection (Fig. 6A) and significantly suppressed M1 factors TNF-α (30-fold), IL-1β (230-fold), IL-6 (116-fold), IL-12p40 (not detected anymore), FcγRI (32-fold), Ciita (18-fold) and CD86 (7-fold) (Fig. 6B). Treatment with Lip-PLP even suppressed M2 factors and only CD163 expression was somewhat upregulated by Lip-PLP (4-fold), suggesting that Lip-PLP inhibits joint inflammation in ICA largely through suppression of M1 macrophages.

## Discussion

In earlier studies we found that a single systemic injection of PLP-containing liposomes very efficiently downregulated joint inflammation and destruction during AIA. In the present study we studied the mechanism of this treatment on macrophage polarization within the inflamed synovium of AIA. During the 7-day course of AIA, we found a strong upregulation of M1 markers in the synovium as measured with micro-array. A single injection of PLP-liposomes strongly suppressed M1 signature in favor of an M2 signature.

The effect of Lip-PLP on gene expression of M1 and M2 markers in the synovium was measured at 24 hours after treatment. At that time-point, Lip-PLP treatment had caused a strong suppression in joint swelling as measured by ^99m^Tc uptake. This rapid suppression of joint swelling is probably mediated by down regulation of oxidants like superoxide radicals, reactive oxygen species (ROS), nitrogen oxygen species (NO) or lipocortin/vasocortin which largely drive vascular permeability and oedema [24]. A recent study showed that corticosteroids are potent inhibitors of superoxide radicals, ROS and NO species in macrophages by reversing induction of iNOS mRNA, NOS activity and NO levels [25]. Although joint swelling was already significantly decreased at day 1 after Lip-PLP treatment, the cellular infiltrate was not significantly changed within the knee joint, thus forming good premises for comparison of genes within the synovium.

PLP-liposomes may be taken up by monocytes and additionally transported to the inflamed joint. However, after intravenous injection of fluorescent PLP-liposomes, no fluorescent monocytes were detected using FACS analysis (data not shown). The small sized (100 nm) unilamellar liposomes used in our study are able to migrate through the blood vessels in the inflamed knee joint which lie just beneath the intimal lining layer. After crossing the endothelium they are taken up by macrophages lying within the thin synovial intimal layer. Macrophages efficiently bind and phagocytose liposomes and *in vitro* no difference in uptake was observed between macrophages with different activation status (M1 versus normal) or between empty liposomes or liposomes filled with glucocorticoids.

The uptake of Lip-PLP by activated macrophages *in vitro* strongly suppresses M1 cytokines TNF-α, IL-6 and IL-12, but stimulates expression of the anti-inflammatory cytokine IL-10. This is in line with a study on activated monocytes by Frankenberger *et al.*, who reported that liposomal methylprednisolone suppressed TNF-α, but stimulated IL-10 production in synergy with LPS activation of human monocytes [26]. Moreover, IL-10 expression was elevated in our *in vivo* experiments compared to naïve mice, but was not suppressed by Lip-PLP *in vitro*. The high IL-10 production could be an important contribution to the anti-inflammatory effects of Lip-PLP as IL-10 and glucocorticoids can work synergistically on the suppression of inflammation during experimental arthritis [27].

*In vitro* uptake of Lip-PLP by M1 macrophages also suppresses the M1 phenotype, as characterized by expression of CD86, and either enhances or maintains expression of M2 genes, thereby skewing these cells into a more M2-like character. This is in line with other studies showing that free glucocorticoids induce M2-like macrophages in human monocytes [12], [28]. A recent study by Varga *et al.* showed that mice treated with corticosteroids induced an anti-inflammatory subset that resembled myeloid derived suppressor cells [12]. Characteristic for M2 macrophages is the expression of CD163, which is a well-recognized marker for anti-inflammatory macrophages in humans and mice [12], [29]. In the present study, Lip-PLP caused an upregulation of CD163 in bone marrow macrophages which was also found to be upregulated in the synovium at day 1 after treatment of experimental arthritis models ICA and AIA (although not statistically significant in the latter). Therefore, this scavenging receptor provides a valuable read-out to determine the anti-inflammatory effect of glucocorticoids on macrophages in models for inflammatory disease. PLP-liposome uptake by M1 macrophages stimulated also other mediators of M2 like TGF-β, IL-1RII and Ym1. Corticosteroids have earlier been shown to be potent inducers of IL1RII in mouse primary activated astrocytes [30].

In contrast with our *in vitro* data, Lip-PLP *in vivo* mainly downregulated M1 but did not enhance the M2 signature. Also, the effects of Lip-PLP on M1 and M2 signature *in vivo* in the synovium were less pronounced. An explanation for that may be that Lip-PLP that was exclusively taken up by a thin layer of lining macrophages and not by macrophages lying at a more distant location, may induce a more focal induction of M2 only within this lining layer. The synovium used for M1/M2 investigation included many macrophages not targeted by liposomes which may dilute the ultimate results for shifting to M2. Earlier studies have shown that synovial macrophages within this thin lining layer drive propagation of synovial inflammation during antigen-induced arthritis. Selective elimination of only lining macrophages by local application of clodronate-containing liposomes in the knee joint during established arthritis almost completely suppressed synovial inflammation within a few days [4]. The lining macrophages form the first layer that meets antigens released from the cartilage or antigens reaching the joint via the blood.

The lining cells may control early joint inflammation by upregulating suppressive molecules. During the first week of AIA, M1 markers in the synovium are highly expressed whereas most M2 markers remain low. Interestingly, a strong upregulation of M2 markers Arg1 and Ym1 was observed during the first days of the AIA which may regulate synovial inflammation during the first phase of AIA. Both genes are specific markers for murine M2 macrophages [31]. Arginase 1 is an enzyme that competes with iNOS for L-arginine and reduces the accumulation of reactive oxygen species (ROS) [32]. The physiological role of Ym1 is not clear but a role in promotion of cytokines is suggested [32]. Expression of Ym1 (but not Arg1) was raised by Lip-PLP treatment of macrophages *in vitro* but also in the synovium after 1 day of treatment of AIA. Ym1 promotes Th2 cytokine expression like IL-4 and IL-13 by inhibiting 12/15 lipoxygenase [33]. These cytokines are expressed during AIA and have been shown to strongly regulate synovial inflammation within this model [34]. In direct response to IL-4 and IL-13, Ym1 is strongly upregulated in murine macrophages in a STAT-6 dependent manner [35] thereby forming a positive feedback loop which may drive further Th2 differentiation.

Unlike Ym1, other mediators of M2 macrophages like IL-10, TGF-β, IL-1RII, CD206 and FIZZ1 remained at the same level and were not altered by Lip-PLP treatment whereas in contrast M1 markers were strongly downregulated. Altogether this suggests that there is no shift towards the M2 as the dominant phenotype within the synovium after treatment with Lip-PLP.

In AIA, we have found evidence of favoring M2 by decreasing M1 markers whereas in the ICA there is more an overall non-specific decrease in M1 and M2 markers. An explanation for this discrepancy may be that under *in vivo* conditions macrophages which have taken up PLP-liposomes meet additional triggers like ICs and T-cells which prevent an effective differentiation towards an M2 status. ICs that drive joint inflammation in ICA can stimulate macrophages into an M1 phenotype by binding to activating FcγR. In the AIA joint, apart from ICs also Th2 cells meet synovial macrophages which produce cytokines like IL-4 and IL-13 which may counteract the IC effects. Previous studies in our lab have shown that overexpression of either IL-4 [36] or IL-13 [37] during ICA strongly diminished joint inflammation and destruction, probably by differentiating macrophages into an M2 phenotype.

Treatment of arthritis with a single systemic injection of PLP-liposomes has been shown to be much more effective than free corticosteroids. This study clearly shows that selective targeting of PLP-liposomes to synovial intimal macrophages strongly suppressed M1 in both arthritis models whereas M2 was lower in ICA and not altered in AIA. Suppression of the M1 signature by liposomal PLP may drive the inflammatory status in the synovium towards a more positive and more efficient treatment for patients suffering from auto-immune disease.
